# Parametric Study of Planetary Milling to Produce Cu-CuO Powders for Pore Formation by Oxide Reduction

**DOI:** 10.3390/ma16155407

**Published:** 2023-08-01

**Authors:** Julian E. Tse Lop Kun, Adam P. Rutherford, Ryan S. Learn, Mark A. Atwater

**Affiliations:** School of Engineering, Liberty University, Lynchburg, VA 24515, USA

**Keywords:** solid-state foaming, porous metals, planetary milling, additive expansion by reducing oxides, copper, copper oxide

## Abstract

Powder-based methods that are used to make porous metals are relatively simple and scalable, and porosity can be controlled by interparticle spacing as well as the addition of a sacrificial template. A relatively new process based on reducing oxides in a metal matrix has been demonstrated to produce microscale porosity *within* individual powder particles and thereby may be used to enhance other powder metal techniques. Templating methods require relatively large quantities of powder, but oxide-reduction feedstock powders have only been produced by small-batch ball milling processes (e.g., 10 s of grams). Planetary ball milling is capable of processing larger quantities of powder (e.g., 100 s of grams) but has significantly different milling characteristics. To successfully apply this technique, it was systematically studied in terms of composition, milling conditions, and the addition of stearic acid to control powder size and morphology along with final porosity. It was found that by controlling basic parameters, such as oxide levels and milling time, a relatively high porosity (25%) and powder percentage (99%) can be achieved in Cu-2 mol% CuO with only 0.035 wt% stearic acid and only 90 min of milling.

## 1. Introduction

The introduction of porosity in metals provides unique lightweight and functional properties, including a high strength-to-weight ratio, energy absorption (crushing, acoustic, etc.), enhanced reactivity for catalysis or electrodes, and low thermal conductivity [1]. As noted in a review of solid-state foaming methods [2], the diversity of approaches and outcomes is wide-ranging, but solid-state foaming has seen limited commercialization because it is often more complex to produce pores in solid materials, and each process is suited to a small group of alloys or applications. Therefore, solid-state foaming provides great potential if the challenges associated with mass production can be overcome.

Powder metal (PM) techniques to achieve porosity can be as straightforward as incomplete sintering (i.e., leaving residual porosity), which is already being applied in AmesPore^®^ filters and SELFOIL^®^ bushings produced by AMES (Sant Feliu de Llobregat, Spain). For higher porosity and specific pore size and morphology control, powder can be distributed on or around a template that is later removed and provides porosity that is often hundreds of microns or millimeters in scale [3]. Gas entrapment, which is often applied to titanium, uses an argon-pressurized container and titanium powder to trap the argon between powder particles during hot isostatic pressing, and post-compaction heating allows expansion of that gas to create pores [4]. These strategies can achieve relatively large-scale components, but the intricacy of the processes can present challenges to low-cost, high-production adaptations. Large components are not always desirable, however, as small-scale porosity and modest dimensions are useful for functional applications such as catalysis or energy storage [5], and although not common, nanoscale porosity can also be produced in powders [6,7].

A commonality among traditional PM methods for metal foams is that they all rely on porosity *between* particles, but porosity can also be introduced *within* particles. The additive expansion by the reduction of oxides (AERO) process mixes oxides into metal particles, thereby creating a metal matrix composite (MMC), which is also an oxide-dispersion-strengthened (ODS) metal. Those ODS powders, either loose or compacted, are then reduced in a hydrogen atmosphere to convert the oxides into microscale and nanoscale pores. Therefore, whether by sacrificial template, gas entrapment, or incomplete sintering, the overall porosity can be increased using AERO feedstock. The need for kilogram quantities of this material presents a challenge, as the ODS powders have only been demonstrated in Gram-scale quantities.

In past research, various parametric studies have been conducted to quantify the milling, compaction, and annealing processes that influence the structure and properties of various alloys. Some of these factors include the ball-to-powder ratio (BPR), the powder composition, the milling speed, the milling time, the milling medium, the pressure applied to create the compact, the annealing temperature, and the annealing time. In early work on the AERO powders, it was found that an average expansion of 37% could be achieved after cryogenically milling ~3 mol% of cupric oxide (CuO) with copper (Cu) powder for 90 min and annealing the compacts at 600 °C for an hour [8]. In a later iteration, Cu 2 mol% CuO milled for 30 min at room temperature and annealed at 800 °C resulted in the greatest porosity (~40%) [9], testifying that the milling conditions have a strong influence on the best processing conditions.

Although these efforts have demonstrated clear potential for low-cost, industrial-scale processing [4,5], no significant progress in throughput has been reported. All previous studies have been conducted using high-energy SPEX milling, which is equipment-limited to ~10 g of powder per run or less. Through planetary milling, as studied in this work, the production of AERO powder can be increased twentyfold, with the direct ability to be increased even further. In this study, we investigated the importance of numerous milling parameters on the powder characteristics and pore formation of Cu-CuO compositions. This study was conducted to evaluate the process-property relationships by varying the oxide levels, milling duration, and surfactant addition to determine if the process can be scaled successfully. The findings indicate that pore formation is maximized at short milling times, while stearic acid is critical to avoid rapid particle agglomeration. The process parameters must be carefully balanced, however, as stearic acid can also limit pore formation.

## 2. Materials and Methods

### 2.1. Powder Processing

High-purity copper (Cu, 99%, <75 μm, Sigma-Aldrich #2007780, St. Louis, MO, USA) was mechanically mixed with cupric oxide (CuO, 98%, <10 μm, Sigma-Aldrich #208841) in varying ratios (1, 2, or 3 mol%) using a Fritsch Pulverisette 5/2 planetary mill (Fritsch GmbH, Idar-Oberstein, Germany) using a mass-based ball-to-powder ratio (BPR) of 5:1. Stearic acid (SA, reagent grade, 95%, Sigma-Aldrich #175366) was added in some milling experiments as the processing control agent (PCA) in order to control powder yield. 500 mL hardened stainless-steel vials and 9.5 mm (3/8 in) diameter, grade 25,440 C stainless steel ball bearings (Salem specialty ball) were used. Powders were handled and stored in a glove box under an argon (Ar) atmosphere, and milling vials containing the powders and the balls were also stored and closed in the Ar atmosphere before milling. Each composition of Cu-CuO was milled at a rotational rate of 300 rpm (10π rad s^−1^) for various milling times (30, 60, 90, or 120 min).

After the milling process, the powders were thoroughly sifted through stainless steel sieves with various mesh spacings: 1 mm, 500 μm, 125 μm, 75 μm, and 38 μm. The weights of the sieved composition were recorded to quantitatively interpret the powder size distribution. Herein, particle sizes under 500 μm were termed “powder”, and the rest were considered “spheres.” The powder percentage of each milled composition is described as the powder mass over the total mass of the milled composition, as shown below:

(1) $$Powder\;Percentage\left(\%\right)=\frac{m_{powder}}{m_{total}}\times 100\%$$

### 2.2. Compacting

Three samples of each milling condition, 5 g each, were uniaxially compressed at room temperature at 1 GPa (Carver Inc., Auto Pellet Press Model 4387, Wabash, IN, USA) and held at that pressure for 1 min in a 12 mm diameter cylindrical tungsten carbide die. The punches and die were thoroughly cleaned with ethanol before use to reduce contamination. The mass and dimensions of each compact were obtained to calculate the geometric density before and after the annealing process. The density presented throughout this paper represents the average for a set of at least three samples with an error of plus or minus one standard deviation from the average.

### 2.3. Annealing

Samples were placed in a 50 mm diameter, single-zone tube furnace (Across International STF1200, Livingston, NJ, USA) that was heated to 600 °C in a 5% H_2_ (bal. Ar) atmosphere with a flow rate of 0.4 L min^−1^. Throughout this research, the annealing temperature was limited to 600 °C as maximum pore production with minimal sintering and densification was desired. While some sintering occurs, this is not to the degree achieved in traditional PM processes, which typically have the goal of full densification. For instance, mechanical and electrical properties are improved in pure and reinforced Cu with sintering at temperatures of 800–900 °C [10], and these temperatures could be used if the performance benefits justify the increase in density. Samples were inserted into the center of the preheated furnace until they reached the desired temperature, and then they were left to anneal and expand for 1 h. The samples were then removed from the furnace and allowed to cool in the same atmosphere. Once cooled, the mass and dimensions of the samples were obtained again to calculate their final density after foaming. In the event of foaming, samples will be lighter in mass and larger in volume due to the foaming process.

In order to quantify the change in porosity, the following formula was used:

(2) $$\Delta p\;\left(\%\right)=p_{a}\;\left(\%\right)-p_{b}\left(\%\right)$$

where Δp is the change in porosity, $p_{b}$ and $p_{a}$ are the porosity before and after annealing respectively. Porosity is defined here as the ratio of the density of the actual sample to the density of the matrix material (copper, $\rho_{Cu}$ = 8.96 g/cm^3^). As such, the percent change in porosity can be expressed with the following formula:

(3) $$\Delta p\;\left(\%\right)=\frac{\rho_{b}-\rho_{a}}{\rho_{Cu}}\times 100\%$$

where $\rho_{b}$ and $\rho_{a}$ are the densities of the sample before and after annealing, respectively. This allows the porosity within particles to be isolated, as the interparticle voids are a function of compaction parameters such as pressure, time, particle size, etc. Again, the foaming of particles is independent of how they are used, so their isolated performance is of greatest interest and most easily measured in compacted form.

### 2.4. Characterization

Powder that was compacted and reduced at 600 °C was then cold-mounted in epoxy, polished by hand using progressively finer abrasive papers to 1200 grit, and then polished using a 1 µm alumina slurry. This was followed by 30 min of vibratory polishing with 0.04 µm silica, after which samples were cleaned with soap and water and rinsed with ethanol. A Keyence VK-X1000 optical microscope (Keyence Corporation of America, Itasca, IL, USA) was used to image the sample surface and assess porosity. Loose powders were imaged using a JEOL JSM-IT100LA scanning electron microscope (SEM, JEOL USA, Inc., Peabody, MA, USA) operated at 20 kV at a working distance of 10 mm and a probe current of 60 (the manufacturer’s setting). The powders were mounted onto carbon tape and then placed on the standard sample stage for the microscope.

## 3. Results

### 3.1. Preliminary Process Investigation

#### 3.1.1. Effects of Milling Time

**Figure 1:**
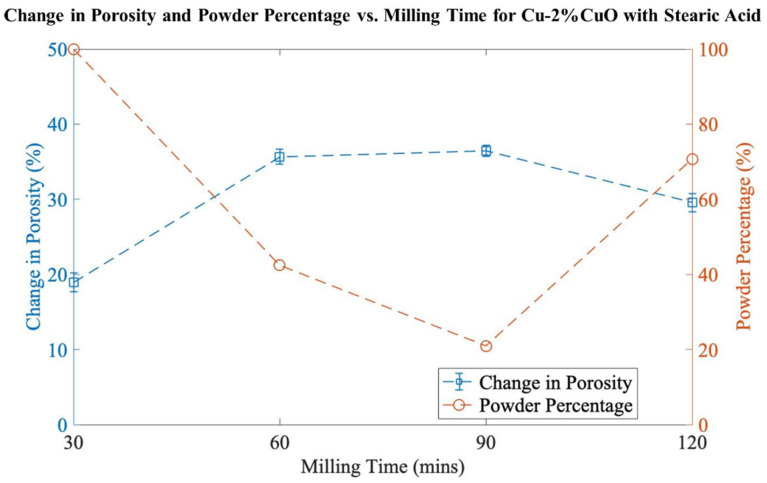
Change in porosity and powder percentage vs. milling time for Cu-2%CuO.

The goal of the preliminary investigation was to identify the most critical factors in the milling process. Based on previous research [8,9], the initial composition chosen was 2 mol% CuO (bal. Cu), and milling durations of 30, 60, 90, or 120 min were applied. Figure 1 presents the effects of milling time on the change in porosity and powder percentage. Ideally, a high percentage of porosity will be combined with a high percentage of powder yield. It can be observed that the change in porosity increases with increasing milling time; however, there is a drastic fall in the powder percentage throughout the first 90 min. The change in porosity is maximized at 36.4%, but this comes with the drawback of having a low powder percentage of 21.0%, with the remaining material being present in large, spheroidal agglomerates. Based on these results, no milling durations longer than 120 min were used.

#### 3.1.2. Effects of Intermittent Cooling Time

**Figure 2:**
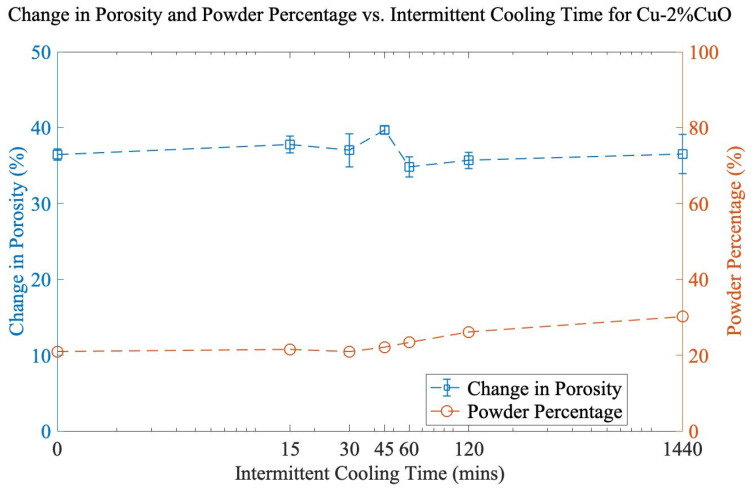
Change in porosity and powder percentage vs. intermittent cooling time for Cu-2%CuO. Total milling time is 90 min for each sample.

The loss of powder yield was addressed as a function of milling time. Since no powder loss occurred after 30 min of milling, three intervals of 30 min were chosen to reach 90 min total (maximum porosity). Various cooling durations (15, 30, 45, 60, 120, and 1440 min) were applied between each 30 min mill. From Figure 2, it can be observed that increasing the amount of cooling time between each 30 min interval has little effect on powder yield and porosity. Total porosity is nearly identical at the extremes of 0 min and 1440 min of cooling time, and the powder percentage rose by only 9% for the longest cooling interval. This indicates that the role of temperature during the milling process is secondary to the cumulative mechanical work of cold welding, and long cooling steps will unnecessarily lower the throughput of material.

#### 3.1.3. Effects of Processing Control Agent

**Figure 3:**
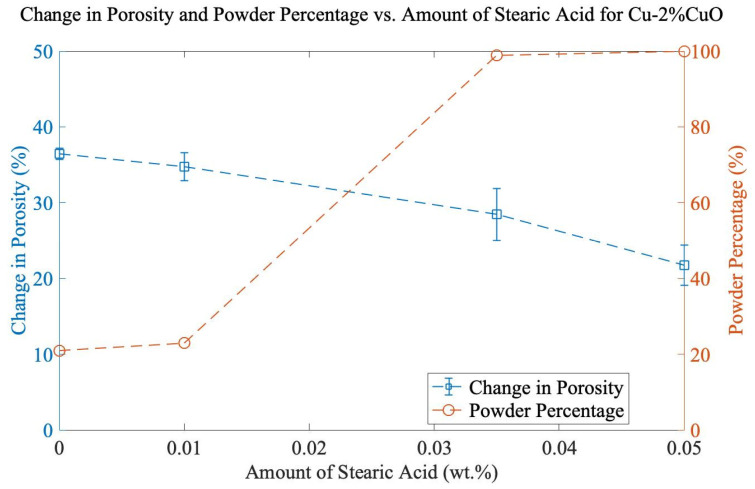
Change in porosity and powder percentage vs. amount of SA for Cu-2%CuO. All samples were milled for 90 min.

In previous research [8,9], cryogenic conditions, or 1 wt% SA, were added to prevent cold welding in the SPEX shaker mill. The motivation for this work was to minimize complexity (no cryomilling) and contamination caused by process control agents (PCAs) while increasing the powder yield. Small amounts of SA (0, 0.01, 0.035, and 0.05 wt.%) were used with Cu-2%CuO milled for 90 min, and it can be seen in Figure 3 that increasing the amount of SA significantly increases the powder percentage but also decreases total porosity. 0.035 wt% SA proved to achieve the best balance of powder and porosity. The 1 wt% addition of SA commonly used in SPEX milling is nearly 30 times higher than applied here, emphasizing the importance of re-evaluating these process parameters.

### 3.2. Primary Investigation of Processing Parameters

Based on the preliminary investigations, the remaining work focused on varying oxide levels (1, 2, or 3 mol% CuO), milling time (30, 60, 90, or 120 min), and SA addition (0 or 0.035 wt%). The goal of this work is to reliably produce powder via planetary milling for creating components by oxide reduction. The optimal conditions in regard to the foaming process have been studied in significant detail via high-energy SPEX milling [8,9]. In particular, a 600 °C foaming temperature over a 1 hr duration was maintained for all samples.

### 3.3. Production of Porous Components

**Figure 4:**
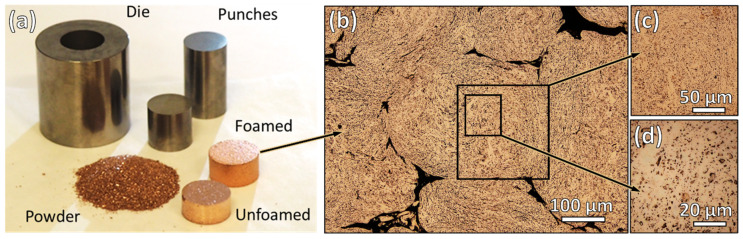
(**a**) Photograph showing various stages of sample processing and (**b**) optical microscope image of polished powder cross-section after foaming (Cu-2%CuO, 0.035 wt% SA, 90 min, 600 °C), including magnified insets of the porosity in (**c**,**d**).

In the foaming process, the powder is pressed and then reduced at an elevated temperature to produce a porous pellet, which also experiences a limited amount of sintering. An example of this is provided in Figure 4. The central aspects under study here are the conditions necessary to produce powder that is suitable for PM component fabrication and maintains a significant degree of porosity. This is dictated by powder size and morphology, as well as the composition and milling duration. As can be seen in Figure 4b–d, the results in the planetary milled powder are consistent with previous findings where micron or sub-micron porosity is produced throughout the volume of individual particles in the compact.

**Figure 5:**
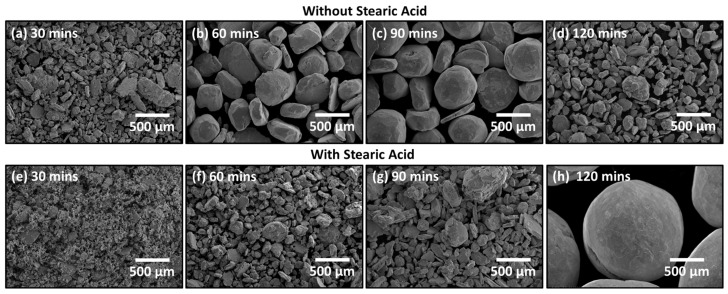
Scanning electron microscope (SEM) imaging of Cu-2%CuO without SA milled for (**a**) 30 min, (**b**) 60 min, (**c**) 90 min, and (**d**) 120 min and with SA milled for (**e**) 30 min, (**f**) 60 min, (**g**) 90 min, and (**h**) 120 min.

**Figure 6:**
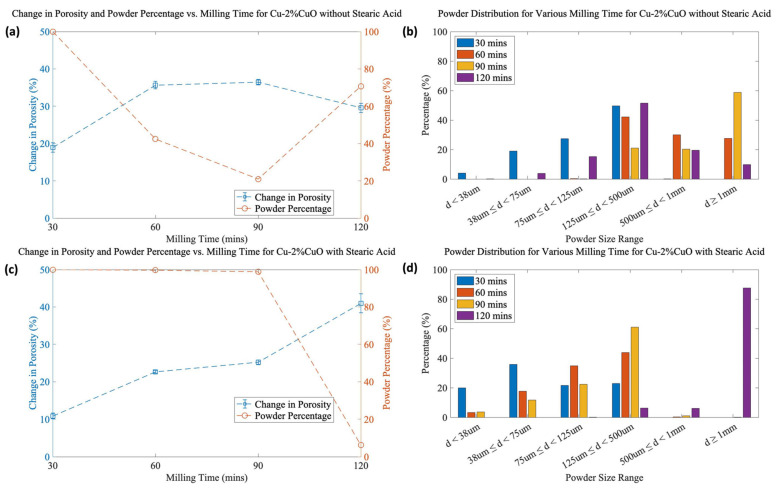
(**a**) Change in porosity, powder percentage, and (**b**) powder size distribution vs. milling time for Cu-2%CuO without SA, and (**c**) change in porosity, powder percentage, and (**d**) powder size distribution with SA.

Powder morphology is important to component fabrication [11], and it was examined using SEM under the milling conditions described above. Figure 5 presents images for Cu-2%CuO at various milling times with and without the addition of stearic acid. Additional figures for 1 mol% CuO and 3 mol% CuO can be found in the Supporting Information. It is generally observed that the powder is irregularly shaped, with a combination of flakes and spheroidal particles. As will be detailed in Figure 6b,d (below), the particle size range changes with time. Whereas 30 min and 120 min of milling exhibit little to no overlap in size range, 60 min and 90 min are quite similar. For instance, Cu-2%CuO milled without SA looks very similar in Figure 5b,c and Figure 6b, which confirms there are significant similarities in their particle sizes, although a greater portion of the largest particles exist after 90 min of milling. Given that SEM imaging samples a rather small quantity of powder from the hundreds of grams produced, the importance of combining it with a quantitative measurement of particle size, especially for relatively coarse powders, is evident. The spheroidal particles increase in proportion after longer milling times. While there may be particular applications where spherical particles are desirable, such as additive manufacturing or metal injection molding, our goal was to produce irregular powders for higher green strength [12].

#### Powder Size and Porosity

Figure 6 presents the change in porosity, the powder percentage, and the powder size distribution for Cu-2%CuO at various milling times with and without SA. Additional figures for 1 mol% CuO and 3 mol% CuO can be found in Supporting Information. It is clear that the SA has two effects: restricting the cold welding of powder and, to a lesser degree, suppressing pore formation. After 120 min of milling, the SA has apparently been consumed in the milling process as the particle size drastically increases, which was observed for all compositions. Likewise, the overall porosity increases for all samples. The “ideal” balance between high porosity and high powder yield must be carefully considered. The factors contributing to these behaviors are discussed in more detail in Section 4.2.

**Figure 7:**
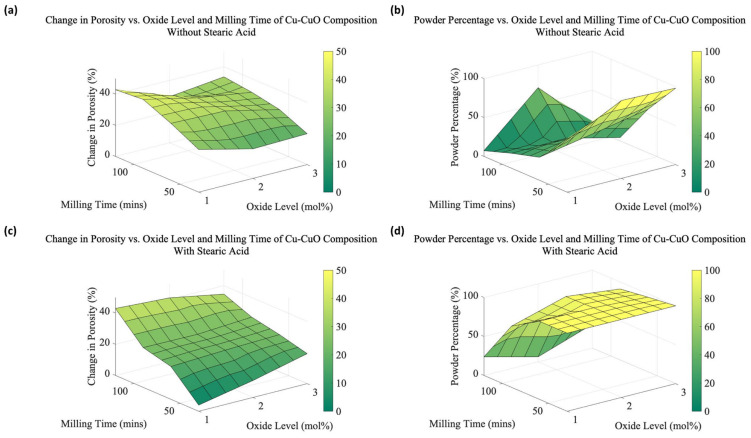
3D Visualization of the change in (**a**) porosity and (**b**) powder percentage vs. milling time and oxide levels for Cu-CuO compositions without SA, and (**c**) porosity and (**d**) powder percentage vs. milling time and oxide levels for Cu-CuO compositions with SA.

From the data collected for all samples, 3D surface plots were created to summarize the trends in average change in porosity and the average powder percentage for various milling times and CuO concentrations (see Figure 7). Figure 7a,b present the expansion and the powder percentage for samples without the addition of SA, respectively, whereas Figure 7c,d show the same factors with SA. Table 1 presents the data collected on the change in porosity and the powder percentage by varying the oxide levels, milling duration, and SA. Figure 7a,c emphasize the difference SA makes, where porosity in lower oxide contents and milling times is severely limited compared to no SA. It is nearly the reverse case in Figure 7d, where powder size is greatly improved except at the longest milling times and lowest oxide content. Powder size without SA is much more complicated and will be discussed in Section 4.2.2.

## 4. Discussion

### 4.1. General Process Factors in Planetary Milling

The two primary goals of this work were to produce a high yield of powder (reduce cold welding) and achieve high porosity. There are many variables that can contribute to the outcome of the milling process, and some may be adjusted easily while others are fixed by the equipment manufacturer. When considering process variables, we focused on the target material system (ingredients being milled) because those factors translate more directly to other milling methods than the milling setup (rpm, ball material, BPR, etc.).

Some other considerations regarding the environment, equipment, and milling media are worth noting briefly, as they may have utility in future research. Active cooling, such as cryogenic milling used in initial AERO feedstock production [13], may further reduce particle size and/or shorten milling duration. Mill parameters are also important. Mio et al. [14] report on the importance of direction and the rotation-to-revolution ratio towards impact energy. The planetary mill used here is designed to produce the maximum impact energy with a revolution radius of 250 mm and a fixed rotation-to-revolution ratio of 2.19:1. Simply changing rotational speed (not ratio) is unlikely to meaningfully reduce particle size as there is less than 1% efficiency in doing so, especially when it comes to ductile materials such as copper [15].

Milling outcomes could also be modified through ball density and the BPR. Higher-density milling media increase the impact energy, which results in more fracturing and smaller powder sizes [16]. For a fixed BPR, smaller balls have lower energy per impact but a greater frequency of impact and greater frictional energy than fewer large balls [17]. Given the unlimited possibilities of tailoring ball charge, it was preferred to use composition to control powder output. By maintaining a relatively low BPR of 5:1, we were able to increase powder output per run.

### 4.2. Primary Investigation of Processing Parameters

#### 4.2.1. Development of Porosity

Pore formation by oxide reduction has been described in detail elsewhere (e.g., [8,9,13]). The central interest of this undertaking was to scale up the process using powder output and volumetric expansion as key indicators of success since the entire component (the pellet) expands in direct proportion to the individual particles. In brief, the pore formation process is related to the formation of steam by CuO reduction and pressurized expansion of the pores. The process of reduction can be represented as follows:

CuO (s) + H_2_ (g) → Cu (s) + H_2_O (g)

Figure 7a,c, and Table 1 summarize the pore-forming performance of the powders produced under varying process parameters. Overall porosity is not directly proportional to any single variable, but there are informative trends. The oxide content is critical to the foaming process, as the formation of porosity depends on the presence of oxide particles within the Cu matrix. It has been determined in previous work that too much CuO can reduce overall porosity as the coalescence and percolation of pores occur too quickly for the *expansion* of the particles to occur, although small pores are still created [8]. The oxide contents used here are all within an acceptable range for significant pore formation, so the porosity differences for specific milling times are generally similar.

The greatest effect on porosity was caused by SA, and the primary mechanism by which it dictates porosity is through particle size. Pores forming in small particles will more quickly connect to the free surfaces and prevent further expansion, and this pressure drop presents a limit to the total pore volume via solid-state foaming by pressurized expansion [18]. The tradeoff between fine powder suitable for component fabrication (more SA) and higher porosity (less SA) is a central challenge to process design. Another side-effect previously observed when using SA with these materials is that a loss of total oxygen content to contribute to the pore-forming process begins early in milling, so it is beneficial to maintain the shortest possible milling time [9].

In the context of commercial PM processing, it is already common to sinter components under reducing atmospheres, so established PM processing is well suited to creating porous Cu components by this method. The extent of sintering at the modest temperature used here will be relatively limited, which is partially chosen to preserve porosity. Even so, other research has demonstrated that reduction during sintering enhances diffusion [19] and provides higher strength than would otherwise be expected at low temperatures. This was recently demonstrated in nanoporous Ni made by oxide reduction, which displayed a specific strength equal to wrought Ni even though it was comprised of powder that had only been heated to 400 °C [7]. Indeed, the mechanical properties of these planetary-milled Cu powders are a topic of interest that will be pursued now that bulk quantities can be produced more readily. Also, a variety of other metals and alloys of industrial importance can be made by the general oxide-reduction process, with oil-impregnated, self-lubricating bushings being a near-term application for Cu-based powders in particular.

#### 4.2.2. Development of Powder Size and Morphology

Throughout the milling process, the material goes through both fracturing and welding. While the collision frequency and energy between ball bearings tend to reach a steady state, friction increases the temperature and causes cold welding between the particles. It has been shown that in the early stages of milling, the temperature rapidly increases, and particle welding is increasingly favored over fracturing. The powder size may also decrease as the work-hardening of the powder causes it to become more brittle and fracturing begins to dominate [17,20,21]. These behaviors can be seen in both Figure 7 and Table 1, where an increase in particle size results in a fall in the powder percentage in nearly all cases. The exception is in the 2% CuO sample, which, based solely on composition and the resulting strengthening of oxide particles, is expected to follow a morphological trend. So, this difference, which was repeatable, is reasonably expected to rely on more than just composition.

Another important factor is early powder morphology. Figure 5 shows that 2% CuO starts with a combination of flakes and spherical particles but shifts to being larger and more spherical over time as the increasing temperature and deformation cause various flake-to-flake, sphere-to-flake, and sphere-to-sphere welding interactions [22]. Cu-1%CuO and Cu-3%CuO (See Supporting Information) show very different morphologies after 30 min of milling. Cu-1%CuO has already experienced extensive agglomeration, while Cu-3%CuO is mostly small flakes. As milling time increases, the Cu-1%CuO powder is ductile enough to resist fracture, and the Cu-3%CuO is comprised of thin flakes, which produce a more intimate structure when agglomerated. The Cu-2%CuO powder is poorly agglomerated as it is neither highly ductile nor comprised of small particles. The “fissures” or “seams” visible in some particles are consistent with this mechanism.

These particle changes during milling and multi-faceted considerations are addressed in other works on the milling of MMCs. For example, when milling AA7075 with TiC nanoparticles, Salur et al. [23] note that the formation of more ductile flakes due to texturing at early milling times eventually breaks up at longer milling times due to mechanical hardening as the grain size reduces, defect density increases, and TiC becomes more uniformly distributed in the Al matrix. Similar results were observed in Mg-Sn [24], where an initial increase in powder size is followed by a significant decrease at longer milling times (up to 12 h) due to hardening and intermetallic formation. It is important to note the difference between the particle size of the matrix and the reinforcement as well. Since the reinforcement phase in an ODS alloy is inherently more brittle, it can break down even as particle size increases, and that can significantly affect material properties, such as hardness, producing increases of as much as 300% [25]. Even the presence of surface irregularities can have significant impacts on functional performance metrics, such as improvements in soft magnetic properties [26], which emphasizes the various considerations that must be simultaneously balanced in powder production.

Even a small percentage of SA added to the Cu-CuO composition was effective in limiting the welding process, causing the particles to remain small during these short-duration milling runs. This is achieved by covering the surface of the powders to limit the direct interactions between the metallic particles [17,20]. After 90 min of milling, most powder compositions with SA retain a high powder percentage (low occurrence of particle welding). Over longer periods, PCAs such as SA are known to break down during milling, and the minute quantity used here may be largely consumed after the first 90 min [27]. As further corroboration of the importance of early-stage powder morphology to agglomerated integrity, the Cu-2%CuO milled with SA starts with fine powder and is then able to sustain large spherical particles, unlike the same composition milled without SA. It is expected that other compositions would eventually breakdown, but the milling times are too short to observe it in this study.

By adding SA, the production of Cu-CuO with both high porosity and high powder can be reliably achieved in only 90 min. Therefore, powder suitable for creating porous components can now be made in larger quantities (100 g per vial vs. 5 g per vial) using planetary milling by balancing the basic parameters of milling time, initial oxide composition, and SA content. The milling time and SA are both used in quantities at least an order of magnitude smaller than are typical in planetary milling. This bodes well for further scaling by related methods and the likelihood of maintaining the cost-effective aspects of the process.

## 5. Conclusions

To increase the production volume of porous copper by oxide reduction, a parametric study was conducted using planetary milling and varying the oxide concentration, milling time, and stearic acid content. These findings are the first to demonstrate the oxide reduction method for pore formation using any process other than a small-scale SPEX shaker mill. By comparing various parameters, processing maps for oxide content, powder yield, and overall porosity were created. The following outcomes were observed:

- As milling time is increased from 30 min to 120 min, the powder percentage falls due to an increase in particle size with cold welding.
- The particles transition from flakes to spheres over time, but as the milling duration increases further, the particles can become more brittle, resulting in a particle size decrease as observed in Cu-2 mol% CuO milled for 120 min.
- Total porosity generally increases with milling time up to 120 min. This is related to particle size increases, such that pores can expand further before coalescing and percolating to the particle surface.
- With only 0.035 wt% stearic acid as a process control agent, cold welding can be almost entirely suppressed during the first 90 min of milling, after which the particle size grows rapidly. Stearic acid has a negative impact on porosity, causing modestly lower overall pore volumes in most cases. Thus, the conditions for producing high powder yield and high porosity are in conflict.
- By balancing the powder yield and pore formation, planetary milling can be successfully applied to produce powders (99% yield) that exhibit micron to submicron pores in the individual particles and can add at least 25% porosity to any powder metal process, such that the overall porosity can be increased. These powders are produced in a very short milling time (90 min) for a relatively high throughput.

## Figures and Tables

**Table 1:** Data for the change in porosity and powder percentage through the influence of varying oxide levels, milling time and SA.

Oxide Levels [mol.%]	Milling Time [mins]	Change in Porosity [%]	Powder Percentage [%]
Without Stearic Acid			
1	30	27.30 ± 0.74	64.54
	60	36.49 ± 1.11	29.37
	90	44.23 ± 2.31	19.20
	120	42.84 ± 0.60	7.24
2	30	18.94 ± 1.24	100.00
	60	35.67 ± 1.01	42.41
	90	36.45 ± 0.75	20.97
	120	29.56 ± 1.21	70.73
3	30	19.88 ± 0.932	98.33
	60	27.78 ± 1.10	55.70
	90	31.27 ± 1.81	4.29
	120	33.51 ± 0.45	5.63
With Stearic Acid			
1	30	3.85 ± 0.34	100.00
	60	20.98 ± 0.69	99.95
	90	25.48 ± 0.49	78.47
	120	42.94 ± 3.95	23.74
2	30	10.83 ± 0.78	100.00
	60	22.67 ± 0.37	99.71
	90	25.21 ± 0.63	98.87
	120	40.98 ± 2.56	6.23
3	30	19.28 ± 1.19	99.91
	60	23.87 ± 0.52	95.80
	90	27.45 ± 2.67	90.98
	120	34.55 ± 1.44	30.43

## Data Availability

The data presented in this study are available on request from the corresponding author.

## Supplementary Materials

The following supporting information can be downloaded at: https://www.mdpi.com/article/10.3390/ma16155407/s1, Figure S1: (a) Change in porosity, powder percentage and (b) powder size distribution vs. milling time for Cu-1%CuO without stearic acid, and (c) change in porosity, powder percentage and (d) powder size distribution with stearic acid. Figure S2: (a) Change in porosity, powder percentage and (b) powder size distribution vs. milling time for Cu-3%CuO without stearic acid, and (c) change in porosity, powder percentage and (d) powder size distribution with stearic acid. Figure S3: Scanning electron microscope (SEM) imaging of Cu-1%CuO without stearic acid milled for (a) 30 min, (b) 60 min, (c) 90 mins, and (d) 120 min and with stearic acid milled for (e) 30 min, (f) 60 min, (g) 90 min, and (h) 120 min. Figure S4: Scanning electron microscope (SEM) imaging of Cu-3%CuO without stearic acid milled for (a) 30 min, (b) 60 min, (c) 90 min, and (d) 120 min and with stearic acid milled for (e) 30 min, (f) 60 min, (g) 90 min, and (h) 120 min.
