# Supplementary material for: Parametric Study of Planetary Milling to Produce Cu-CuO Powders for Pore Formation by Oxide Reduction
Source: Materials (Basel). 2023 Aug 1;16(15):5407. doi: 10.3390/ma16155407 (PMC10420238; doi:10.3390/ma16155407)
Supplement: Supplementary file 1 [file materials-16-05407-s001.zip › materials-2509164-supplementary.pdf]

Supporting Information

Parametric Study of Planetary Milling to Produce Cu-CuO Powders for Pore Formation by Oxide Reduction

Julian E. Tse Lop Kun, Adam P. Rutherford, Ryan S. Learn and Mark A. Atwater *

School of Engineering, Liberty University, 1971 University Blvd, Lynchburg, VA 24515, USA;
jtselopkun@liberty.edu (J.E.T.L.K.); aprutherford@liberty.edu (A.P.R.); rslearn@liberty.edu (R.S.L.)
* Correspondence: matwater1@liberty.edu

Additional data for Cu-1%CuO and Cu-3%CuO are presented here. The powder size, porosity, and morphology are presented in the following figures.

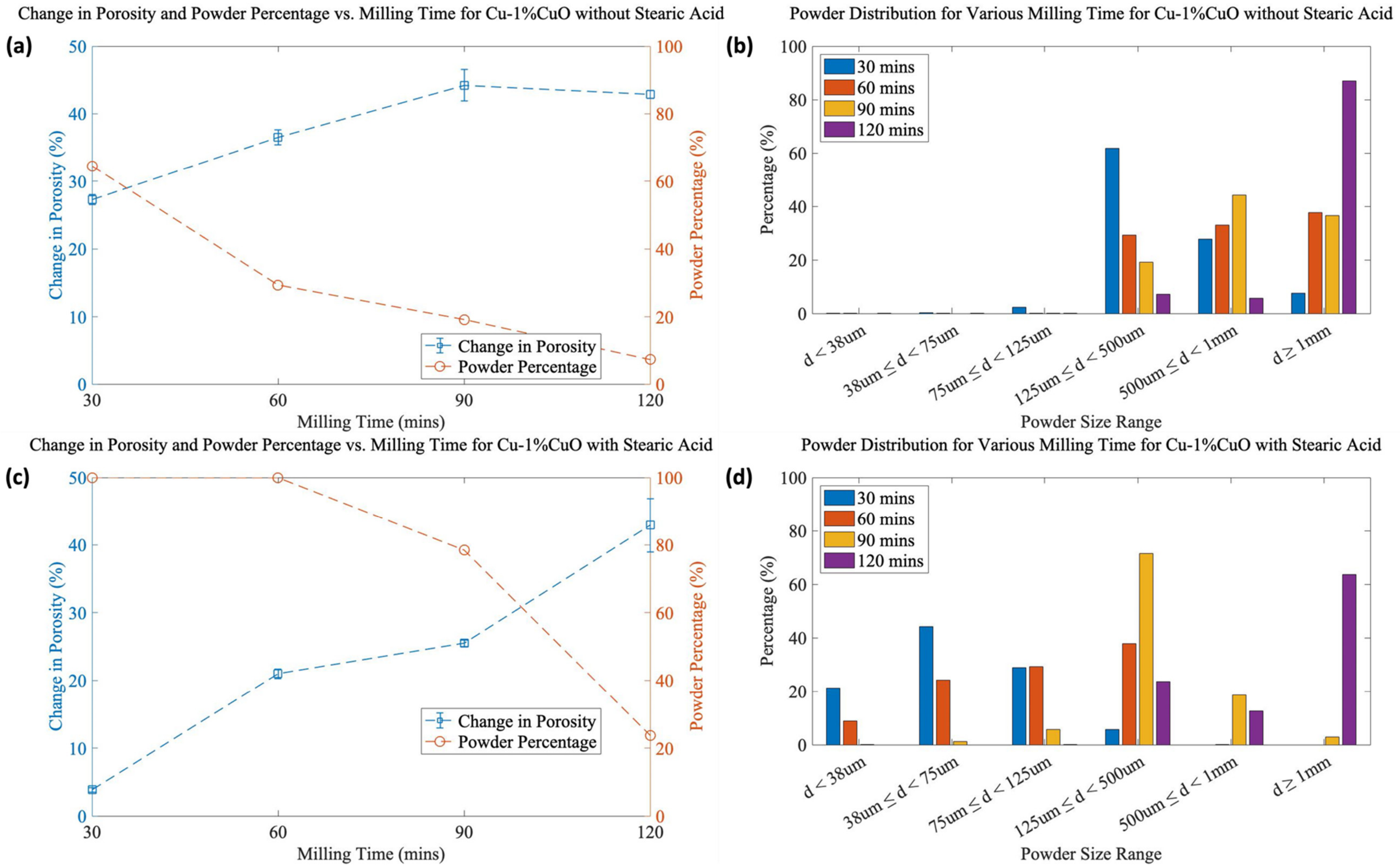

Figure S1. (a) Change in porosity, powder percentage and (b) powder size distribution vs. milling time for Cu-1%CuO without stearic acid, and (c) change in porosity, powder percentage and (d) powder size distribution with stearic acid.

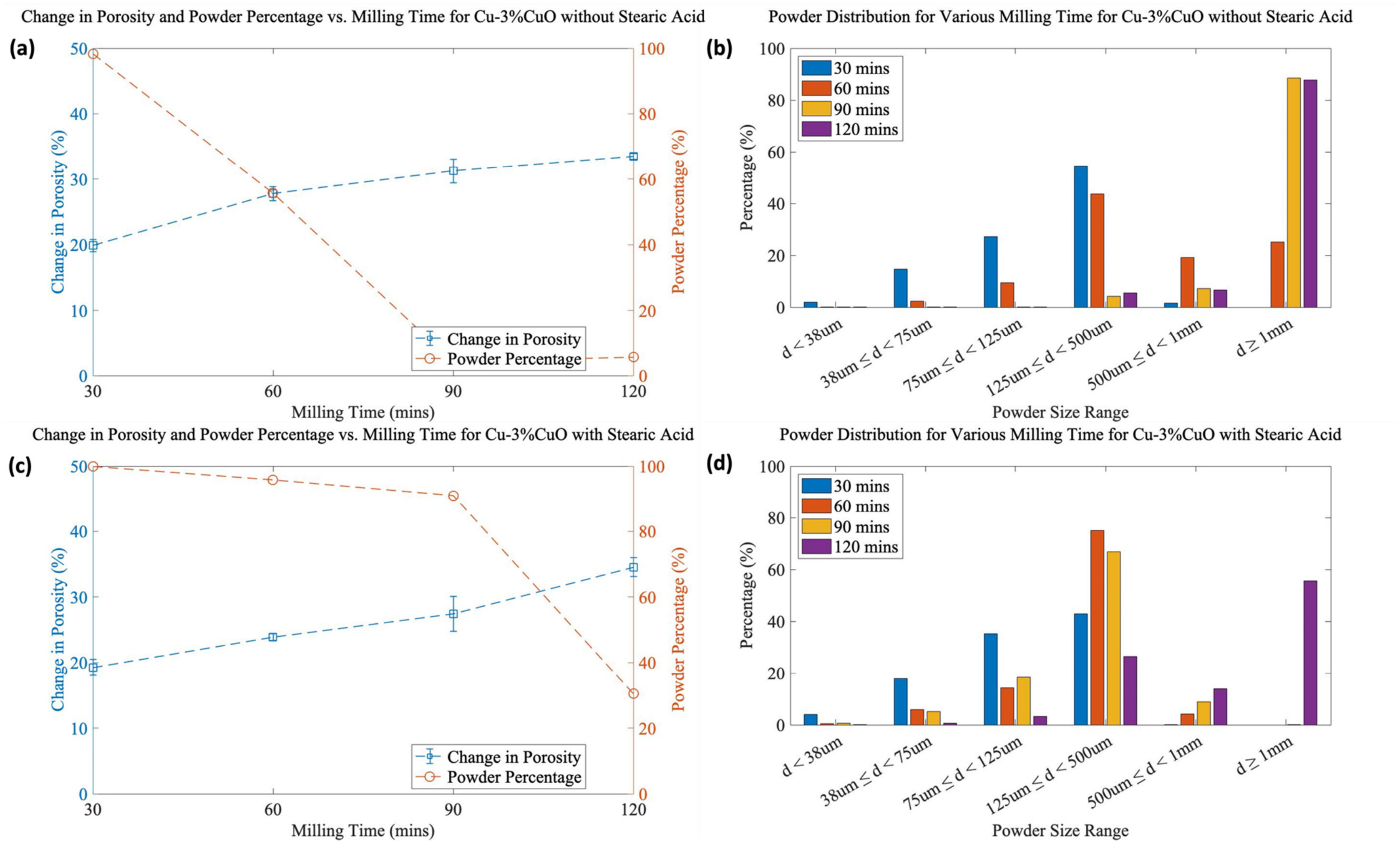

Figure S2. (a) Change in porosity, powder percentage and (b) powder size distribution vs. milling time for Cu-3%CuO without stearic acid, and (c) change in porosity, powder percentage and (d) powder size distribution with stearic acid.

Powder Morphology for Various Milling Time and Various Compositions

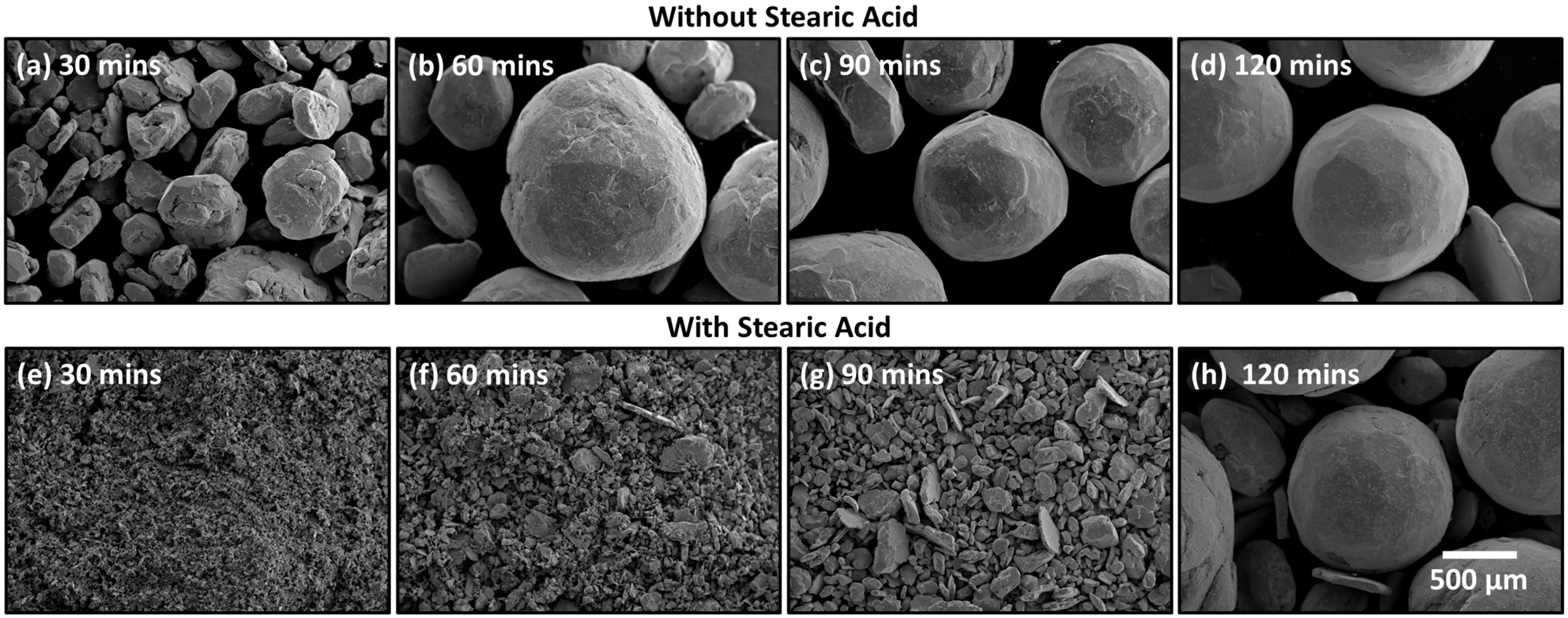

Figure S3. Scanning electron microscope (SEM) imaging of Cu-1%CuO without stearic acid milled for (a) 30 min, (b) 60 min, (c) 90 mins, and (d) 120 min and with stearic acid milled for (e) 30 min, (f) 60 min, (g) 90 min, and (h) 120 min.

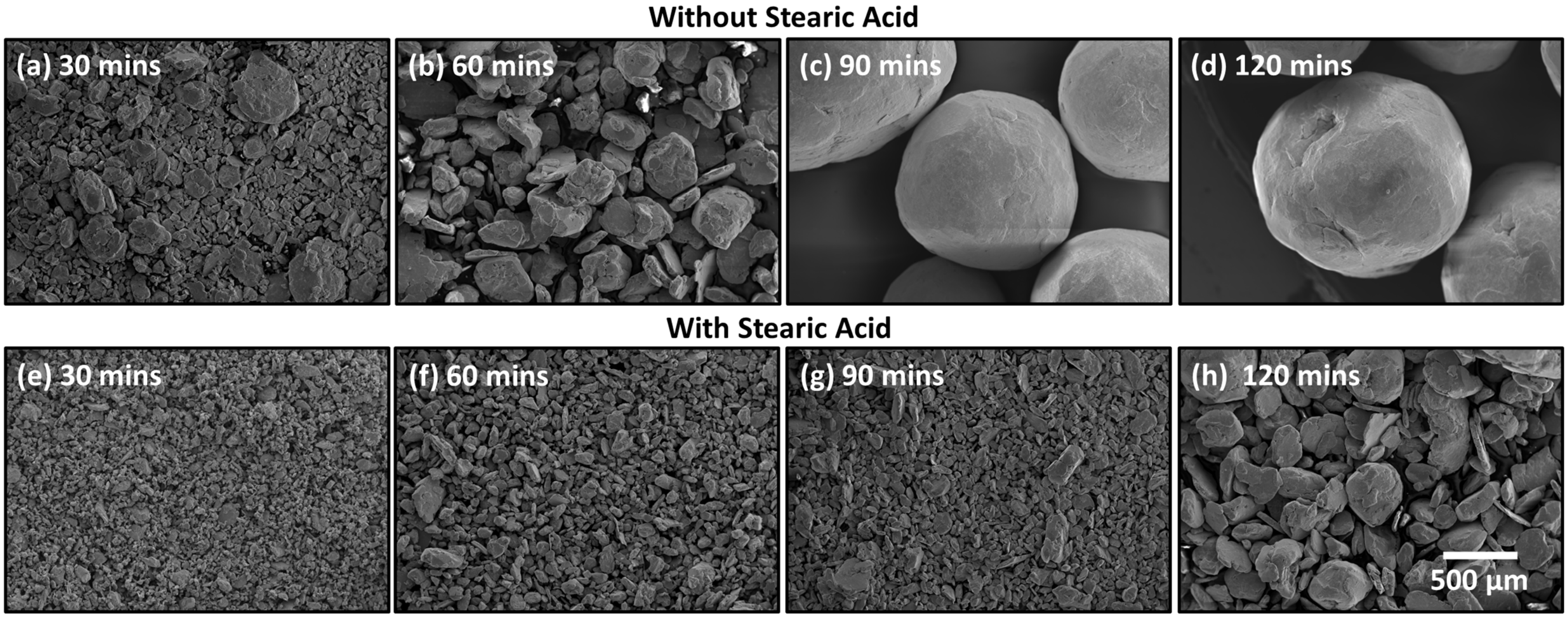

Figure S4. Scanning electron microscope (SEM) imaging of Cu-3%CuO without stearic acid milled for (a) 30 min, (b) 60 min, (c) 90 min, and (d) 120 min and with stearic acid milled for (e) 30 min, (f) 60 min, (g) 90 min, and (h) 120 min.
